# Low Level of Advanced Glycation End Products in Serum of Patients with Allergic Rhinitis and Chronic Epstein-Barr Virus Infection at Different Stages of Virus Persistence

**DOI:** 10.1155/2022/4363927

**Published:** 2022-11-11

**Authors:** Svitlana Zubchenko, Iryna Kril, Halyna Potemkina, Anna Havrylyuk, Aleksandra Kuzan, Andrzej Gamian, Valentyna V. Chopyak

**Affiliations:** ^1^Danylo Halytsky Lviv National Medical University, Lviv, Ukraine; ^2^Department of Biochemistry and Immunochemistry, Wroclaw Medical University, Wroclaw, Poland; ^3^Hirszfeld Institute of Immunology and Experimental Therapy, Polish Academy of Sciences, Wroclaw, Poland

## Abstract

Advanced glycation end products (AGEs) are formed in a nonenzymatic reaction of the reducing sugars with amino groups of proteins, lipids, and nucleic acids of different tissues and body fluids. A relatively small number of studies have been conducted on the role of AGEs in allergic inflammation. In this study, patients with allergic rhinitis (AR) were examined for the presence of Epstein-Barr virus and the content of fluorescent and nonfluorescent AGEs. We have also determined the level of a unique epitope (AGE10) which was recently identified in human serum using monoclonal antibodies against synthetic melibiose-derived AGE (MAGE). The levels of AGE10 determined with an immunoenzymatic method revealed no significant difference in the patients' blood with intermittent AR and chronic EBV persistence in the active and latent phases. It has been shown that there is a statistically significantly smaller amount of AGEs and pentosidine in groups of patients, both with and without viremia, than in healthy subjects. In turn, higher levels of immune complexes than of AGE10 were detected in the groups of patients, in contrast to the control group, which had lower levels of complexes than AGE10 concentration. In patients with active infection, there is even more complexes than of noncomplexed AGE10 antigen. The lower level of AGE in allergic rhinitis patient sera may also be due, besides complexes, to allergic inflammation continuously activating the cells, which effectively remove glycation products from the body.

## 1. Introduction

Glycation is a nonenzymatic process in which reducing sugars react with amino groups of proteins, lipids, and nucleic acids to form a heterogeneous group of molecules known as advanced glycation end products (AGEs) [1–5]. The process takes place in different tissues and body fluids. AGEs play an immunoregulatory role in the body; however, in pathological conditions they can activate receptors for advanced glycation end products (RAGEs) and cause a prolonged inflammatory process [6, 7]. Previous studies revealed the role of AGEs in immunogenicity and the development of several metabolic disorders, like, rheumatoid arthritis, cancer, and diabetes mellitus [8–12]. A relatively small number of studies have been conducted on the role of AGEs in allergic inflammation. A key role in the pathogenesis of allergic disease is a change in the immune response to Th2 lymphocyte switching with the anti-inflammatory cytokine production, and both are involved in the formation of the allergic inflammatory process. It is known that the presence of chronic infections, in particular those caused by human herpesvirus type 4 (Epstein-Barr virus, EBV), can be a trigger of pathological disorders, when the poor or low immune response is observed. The main mechanisms that inhibit viral replication are cellular and humoral immunity, while antiviral protection is supported by Th1-lymphocytes and the corresponding proinflammatory cytokines [13]. In this way, it can be suggested that the alteration of the immune response observed in chronic allergic inflammation may be supported by the presence of a chronic infection and affect AGE production.

Recently we have reported on the synthetic melibiose-derived glycation product MAGE, which mimics a unique epitope present in human and animal tissues [14]. This MAGE product was synthesized in anhydrous conditions, different from the classic synthesis in water solution. The physiological serum epitope called AGE10 was determined with ELISA using anti-MAGE monoclonal antibody [14, 15]. The AGE10 epitope is formed in physiological conditions most likely in unknown pathways of biosynthesis of glycation products. The MAGE cross-reactive autoantibodies were detected in patients with diabetes [14, 15].

The aim of the study was to evaluate the AGE levels in allergic rhinitis patients and the possible connection with chronic EВV infection in different stages of virus persistence.

## 2. Materials and Methods

### 2.1. Patients

A total of 238 patients with allergic rhinitis (AR) were examined in the Department of Clinical Immunology and Allergology of Danylo Halytsky Lviv National Medical University in 2017-2019. Twenty-five patients (14 males (56%) and 11 females (44%), mean aged 32.7 ± 3.2 yrs.) with an intermittent AR were included in the investigation study. Sensitization to the pollen of different plants and verified chronic persistence of EBV was only observed in the patient groups. Nasal symptoms were evaluated using a visual analog scale (VAS) by patients and were classified in accordance with the Allergic Rhinitis and its Impact on Asthma (ARIA) guideline [16]. Patients had not been treated before the study. All patients during the previous pollen season (March-October) had taken topical corticosteroids to control their symptoms. At the time of the study, patients did not receive nasal corticosteroids or antihistamines, although they used nasal saline irrigation if necessary. Twenty healthy blood donor sex and age matched were involved as a control group. Participants gave their written informed consent, and the local ethics committee approved the study.

#### 2.1.1. Inclusion Criteria of the Patients

- Patients of both sexes, age 18-60 years
- Confirmed diagnosis of intermittent AR according to Allergic Rhinitis and its Impact on Asthma (ARIA) criteria
- Patients at the time of examination did not receive nasal corticosteroids or antihistamines
- EBV DNA (in 3 environments—blood, saliva, and mucous)—one of the indicators is positive
- For women of reproductive age—a negative pregnancy test result
- Informed written consent of the patient to participate in the study

#### 2.1.2. Exclusion Criteria of the Patients

- Pregnancy
- EBV as a manifestation of opportunistic infection in HIV infection
- Treatment with immunotropic drugs (immunoglobulins, interferons, interferon inducers, and other drugs that stimulate T and B links of cellular immunity and phagocytosis), within less than 30 days from the time of randomization
- Alcohol or drug abuse
- Any accompanying decompensated diseases or acute conditions, the presence of which, in the opinion of the researcher, can significantly affect the results of the study
- The presence of clinically significant deviations of laboratory indicators that may affect the results of the study
- Participation in any other clinical trial within the last month

### 2.2. Skin Prick Test (SPT) Method

SPTs for the triggering respiratory allergens were performed by standard technique on the palmar surface of the forearm. In the study, we used extracts produced by Inmunotek (Madrid, Spain). The study used commercially available extracts of pollen allergens. Histamine dihydrochloride (1 mg/mL) and 50% glycerosaline were served as the positive and negative control accordingly. SPTs were evaluated within fifteen minutes. A positive skin response was defined as the presence of a wheal with a mean diameter of at least 3 millimeters (mm) greater than that elicited by the negative control accompanied by erythema.

### 2.3. Serum Total IgE (tIgE)

Serum total IgE (tIgE) was determined using a Total IgE-ELISA kit (IBL international GmbH, Germany) according to the manufacturer's instruction. Results for serum total IgE were expressed in IU/mL. Briefly, 10 *μ*L of the undiluted samples and the ready-to-use standards together with 200 *μ*L of the conjugate were introduced to the well plates with the immobilized antigen (specific anti-IgE antibodies). The plate was incubated at room temperature for 30 minutes. The plate was washed with 300 *μ*L of diluted washing solution. Next, 100 *μ*L of the ready-to-use substrate was added to the wells at room temperature and incubated in the dark for 15 minutes. The stop solution was then added. The intensity of the color reaction was directly proportional to the amount of total IgE in the sample. Then the absorption at 450 nm was measured (optionally reference wavelength of 620 nm). The mean values for the measured absorptions are calculated after subtracting the substrate blank value. The difference between the single values should not exceed 10%.

### 2.4. Specific IgE

The Polycheck® (Biocheck GmbH, Germany) is a multiple allergosorbent test for the quantitative measurement of allergen-specific IgE in the serum. Twenty lines of relevant allergens are coated together with five lines of calibrators on a carrier membrane, which is located in the well of the Polycheck cassette. The twenty lines represent the following respiratory allergens: pollen of plants, pets, molds, and home dust mite (HDM). Briefly, during incubation with patient's serum in the well at room temperature (RT), the allergen-specific IgE was bound to the corresponding allergens. Monoclonal ligand-labelled anti-IgE antibodies were incubated in the well cassette. Enzyme-labelled antiligand was added to the Polycheck cassettes, incubated, and bound to the immune complexes. The substrate solution was added and incubated in the dark. The color intensity of the lines was proportional to the respective allergen-specific IgE concentration in the patient's serum. The cassettes were interpreted with the Polycheck imaging software (BIS). Results were quantitatively reported in international units (kU IgE/L).

### 2.5. Determination of IgG/IgM to Capsid Antigen of Epstein-Barr Virus

The determination of the human anti-Epstein-Barr virus IgG (ab108730) and IgM (ab108732) was performed using ELISA Kits (Abcam, UK) according to the manufacturer's instructions. The patients' serum served as research material. The samples and control specimens were incubated in well plates with an EBV-immobilized antigen. Unbound serum components were washed away. Human IgG/IgM antibody was detected using conjugate labeled with horseradish peroxidase. After that, the plates were washed and TMB was added to the wells. The reaction was stopped by the addition of STOP Solution, and the optical density was measured at 450 nm using a Sunrise microplate reader (Tecan, Austria). The color intensity was proportional to the amount of antibodies to the capsid EBV antigens in the test sample.

### 2.6. EBV Detection by PCR

Detection of EBV in blood, saliva, and oropharynx was performed by PCR using AmpliSens® EBV-screen/monitor-FRT PCR kit with “Rotor-Gene 6000” (Corbett Research, Australia) and was based on the amplification of pathogen genome-specific region using special EBV primers. Briefly, the DNA extraction of each clinical sample was carried out in the presence of internal control. The PCR-mix and polymerase (TaqF) mixture was prepared and vortexed without forming foam. The prepared PCR-mix mixed with DNA obtained at the DNA extraction. The results were interpreted by the software of the used instrument on the basis of the fluorescence curve crossing (or not crossing) on the threshold line.

### 2.7. Determination of AGE10

Advanced glycation end products AGE10 were determined with ELISA according to the published procedure [14, 15, 17–20], using mouse anti-MAGE monoclonal antibody, obtained in the Institute of Immunology and Experimental Therapy, Wroclaw. Briefly, samples of serum were pretreated with proteinase K (Sigma) to release AGE10 prior their use as inhibitors in the test. Proteinase K was inactivated by denaturation (120°C, 10′). Concentrations of the synthetic low molecular-mass MAGE (LMW-MAGE) standard were increased to give a calibration curve in the range 0-1.6 *μ*g. The sorption plate (PolySorp, Nunc) was coated with the high molecular-mass MAGE (HMW-MAGE) product corresponding to the antigen to which the antibodies were directed. The MAGEs were obtained by high-temperature microwave synthesis and isolated by liquid chromatography (column with HW-55S gel in 0.01 M ammonium acetate buffer) [14, 15, 17]. Monoclonal mouse antibodies IgE anti-MAGE in culture supernatant were added to sera or LMW-MAGE standard and applied to a sorption plate. After 2.5 hours of incubation at RT, the plate wells were washed and treated with a solution of secondary–polyclonal antibody to mouse IgE (Fc specific)–peroxidase (HRP) (OriGene, 1 : 6,000) at a dilution of 1 : 10,000, and after 2 hours, a colorimetric reaction with OPD was carried out. A control test in which there was no reaction with primary antibodies was done. Based on standard curves, the amounts of AGE10 in serum were calculated and then converted to 1 mL of serum. Additionally, fluorescent AGEs were determined with measurements of fluorescence at 335/385 nm of excitation/emission, specific for pentosidine and at 370/440 nm of excitation/emission, specific for total advanced glycation end products [21].

### 2.8. Determination of Immune Complexes (IC)

The method of Turk et al. [22] was applied for recovering ICs from serum, which were determined as previous [18]. Wells of a 96-well microtiter plate (Nunc) were coated with 5 *μ*g of HMW-MAGE in 100 *μ*L of 0.2 M carbonate buffer, pH 9.6. The wells were blocked with 5% skim milk and washed 3 times with 250 *μ*L of PBS-T (3.6 mM Na_2_HPO_4_, 1.4 mM NaH_2_PO_4_, 150 mM NaCl; 0.05% Tween, pH 7.4). Then, the proteinase K-treated samples of isolated immune complexes were added to monoclonal IgE anti-MAGE antibodies diluted with PBS (50 *μ*L of sample with 25 *μ*L of enzyme and 225 *μ*L of antibodies), and the mixtures were incubated for 45 minutes at room temperature and then applied to the washed sorption wells (100 *μ*L/well). After 2-hour incubation and threefold washing with 250 *μ*L of PBS-T, the wells were treated with goat horseradish peroxidase-conjugated antimouse IgE (1 : 6000, OriGene, Rockville, MD, USA) at room temperature for 1.5 hour. Then, after threefold washing with 250 *μ*L of PBS-T, the bound antibodies were detected in reaction with *o*-phenylenediamine (Sigma), and absorbance at 450 nm was read with the Epoch microplate reader (BioTek Instruments). AGE10 concentration in immune complexes was calculated on the basis of the standard curve obtained as above for the determination of AGE10.

### 2.9. Statistical Analysis

The results were statistically evaluated by Student's *t*-test. Data are presented as arithmetic mean (*M*) and standard deviation (*m*). Differences at *p* < 0.05 (95.5%) were considered as significant. The analysis of the obtained results was performed using “STATISTICA 10”.

## 3. Results

SPTs were used to detect the sensitization to grass mix (13 patients), Timothy grass (10 patients), silver birch (7 patients), weed mix (6 patients), mugwort (4 patients), common ragweed (2 patients), alder (2 patients), and European ash (1 patient). Only seven patients were sensitive to one allergen. The results of the specific IgE determination with ELISA confirmed sensitization to pollen allergens only, but 6 of all were monosensitized. Increased levels of IgE–114-865 kU/L were revealed in all patients. The active phase of chronic EBV infection was identified by the presence of EBV DNA (number of EBV-DNA copies—10^3^-10^7^/ml)—in the blood, saliva, and the posterior wall of the pharynx and increased titers of specific antibodies EBV-VCA-IgG and EBNA-IgG in 5-10 folds. No DNA virus was detected in the latent phase of chronic EBV infection during PCR analysis but increased titer of specific EBNA-IgG and EBV-VCA-IgG in peripheral blood was observed. No EBV DNA positive results were detected only in the blood of patients.

Twenty-five patients based on general clinical, laboratory, and instrumental methods, specific allergy tests and molecular genetic investigations were divided to the groups—15 patients (group 1, EBV+) with intermittent AR with an active phase of chronic EBV infection (PCR “+” in saliva and/or mucosa) and 10 patients (group 2, EBV-) with intermittent AR and latent form of EBV infection. A group of 20 healthy blood donor sex and age matched was used as a control group.

**Figure 1:**
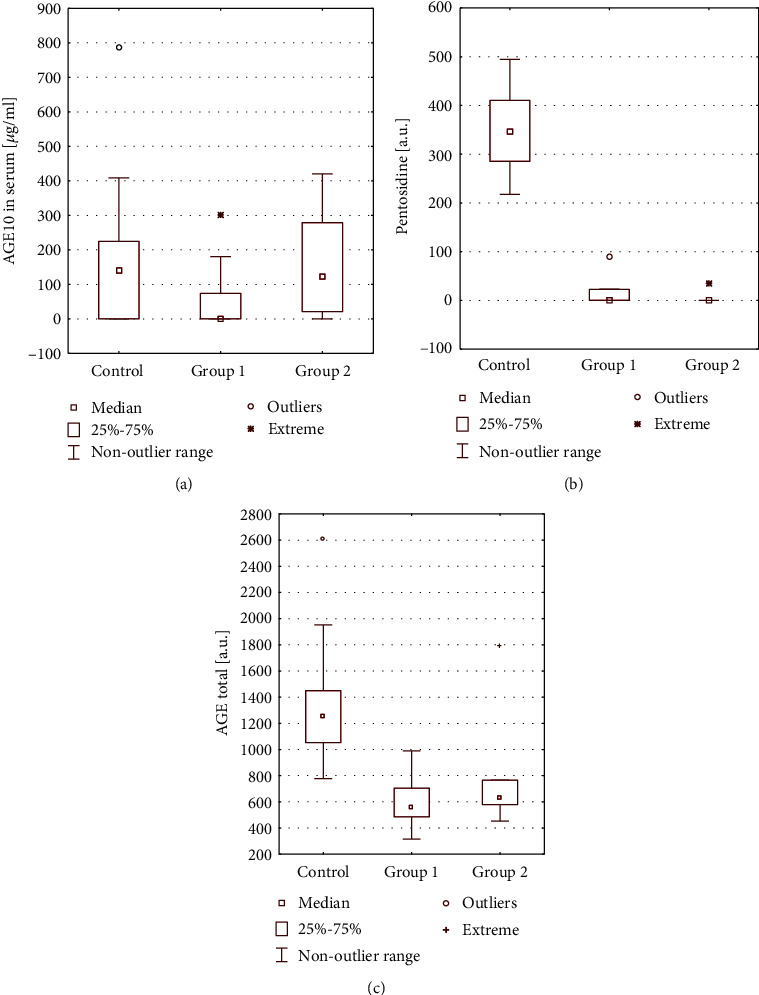
Comparison of AGE level between a group of patients with allergic rhinitis groups and a control group, determined with immunochemical method (a), fluorescence measurement at 335/385 nm for pentosidine (b), and at 370/440 nm for total fluorescent AGE (c) of excitation/emission, respectively.

Using an immunochemical method with MoAb (Figure 1(a)) we found that there was no statistical difference between AGE10 levels in group 1 patients (EBV+) and the control group (*p* = 0.089), between group 2 (EBV-) and the control group (*p* = 0.170), and between group 1 and group 2 (*p* = 0.168). The presence of EBV infection in active or latent phases did not affect the levels of AGE10. Different results were obtained when fluorescence determinations were performed in two different excitation/emission measurements. In both groups of patients, fluorescent AGEs were in significantly lower amounts than in healthy controls (Figures 1(b) and 1(c)). We revealed that the levels of pentosidine type of AGE (Figure 1(b)) were significantly decreased both in patients of group 1 compared to controls (*p* < 0.0001) and in group 2 in relation to controls (*p* < 0.0001), but no statistically significant differences were found between groups 1 and 2 (*p* = 0.618). A very low level of pentosidine in both patient groups was compared to the physiological level of the control group. Next, we found that levels of total fluorescent AGEs (Figure 1(c)) were significantly reduced in patients of group 1 when compared to the controls (*p* < 0.0001) and in group 2 in relation to the controls (*p* < 0.047), but no statistically significant differences were found between groups 1 and 2 (*p* = 0.173).

The results show that AGE10 levels in patients with intermittent AR were not significantly different from those of healthy subjects with their physiological level, measured with an immunochemical method. However, though not statistically significant, the levels of AGE10 in both patient groups had a tendency to be lower than in the healthy group. This tendency could be seen especially in group 1 (with an active infection, EBV+). Regarding fluorescent AGEs, their levels were lower in patients of both groups compared to controls, with a predominantly lower level of total fluorescent AGEs in group 1 (EBV+). It should be noted that our studied AGE10 is different from classical advanced glycation end products [15] .

**Figure 2:**
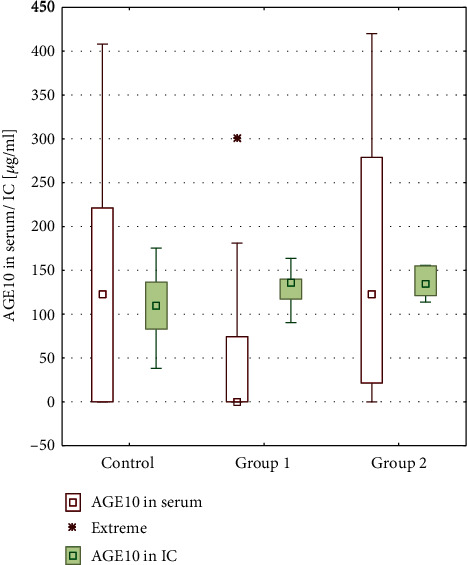
Immune complexes AGE10-anti-AGE10 in patients' sera. For comparison, free noncomplexed AGE10 is shown in the blank square.

In order to find the reason why the studied compounds are below control levels, we have determined the specific immune complexes AGE10-anti-AGE10 in studied sera. The experiments revealed that levels of immune complexes in the sera of patients from both patient groups were higher than in the control group. The amount of immune complexes in the sera of patients from both groups is higher than that of noncomplexed AGE10, although not to a statistically significant degree, whereas in the control group the level of serum immune complexes is lower than free AGE10 concentration (Figure 2). This experiment indicates that low amounts of glycation products in the patients' sera are related to the increased amounts of immune complexes. In patients with an active infection, there are more complexes than of non-complexed-free AGE10 antigen (Table 1). This might be important when to consider immune complexes as severe pathogenic factors. The increased ratio of immune complexes in patient sera, especially in active phase, might explain the mechanism of the disease.

The matrix of correlations prepared for all parameters shows statistically significant correlations. AGE10 in IC negatively correlates with serum AGE10 (*r* = −0.3901, *p* = 0.023), and serum AGE10 positively correlates with total AGE determined with the fluorescent method (*r* = 0.5019, *p* = 0.02). Pentosidine correlates with total AGE measured with fluorescence (*r* = 0.6951, *p* = 0.000). The correlations are presented in Table 1.

## 4. Discussion

It is known that reactive oxygen species (ROS) and reactive carbonyl species (RCS) are involved in the nonenzymatic chemical processes of glycation. These processes lead to the formation of a variety of intermediates and end products [12, 23]. Covalent bond formation between the carbonyl and the amino groups of biomolecules is the initial stage of the nonenzymatic process, which involves amino groups of amino acids, proteins, amino phospholipids, nucleic acids, etc. [24]. However, the direction and nature of further transformations depend on the type of compound containing the active carbonyl group [25]. Glycation concerns not only reducing carbohydrates of the body, but also substances of exogenous nature–industrial air pollutant, tobacco smoke, cosmetic and pharmaceutical products, some foodstuffs, etc. [26–28]. Relatively stable AGEs are produced and accumulated in the cells [27] when the AGE formation processes prevail over their degradation. This is due to the induction of lipid peroxidation (LPO) and the reduction of antioxidant levels. Moreover, oxidative stress can enhance inflammation and tissue damage by enhancing the synthesis of proinflammatory cytokines and altering enzymatic functions. Oxidative stress occurs in many allergic and immunological disorders and in asthma patients [29–31]. Some researchers suggest that oxidative stress in AR patients plays the same role as in asthma patients [32].

A study of Li et al. [33] revealed that enhanced LPO levels in blood and nasal mucosa are in sensitized mice with experimental AR. And conversely, it was found that the levels of antioxidant enzymes–superoxide dismutase (SOD), catalase (CAT), and glutathione peroxidase (GSH-Px) as well as total antioxidant capacity (ТАОС) and glutathione level were significantly decreased in mice with experimental AR compared to mice of the control group [33].

An earlier study by Ogasawara et al. [34] of patients with AR induced by dust mite allergens revealed an increased levels of hydrogen peroxide (H_2_O_2_) released by eosinophils. Hydrogen peroxide is toxic because it has the ability to form free radicals in biological systems. These authors also demonstrated that the number and phagocytic activity of eosinophils were increased in the peripheral blood of these patients, especially in the group of patients with high IgE levels. Therefore, the authors conclude that the release of H_2_O_2_ by eosinophils is an important sign of tissue damage and the severity of the allergic reaction [34]. It was also demonstrated that there were decreased antioxidant levels in the serum of children with bronchiolitis [35]. The same results appear in Bakkeheim et al. [36] investigation where decreased levels of a major serum antioxidant albumin were found especially in schoolchildren with asthma and AR, which correlated with FeNO elevation (a marker of allergic inflammation in asthma) [36].

There are published data that AGEs can be a biochemical marker of oxidative stress and inflammation in some diseases and their complications [37–39]. However, some researchers did not find the presence of oxidative stress markers in patients with AR, because the antioxidant protection and LPO markers were not statistically different between the patient group and the control group [40]. Jiao et al. [41] concluded that oxidative stress is involved in the pathophysiology of AR, but the key marker of allergic inflammatory is induced NOS [41]. Regarding AGEs associated with EBV, there are data indicating that latent membrane protein 1 (LMP1) induce RAGE which is considered an EBV-oncoprotein, and in patients with nasopharyngeal carcinoma this interaction contributed to angiogenesis [42] and metastasis [43].

In our study of the patients' blood with intermittent AR and chronic EBV infection at different stages of persistence, the AGE levels, as determined by the immunoenzymatic method, were not statistically different from those of healthy subjects. It should be noted that blood sampling for the AGE study was performed in the winter, during the intermittent AR remission period. In this period there is no mucosal contact with pollen allergens, which may act as glycating agents that initiate a nonenzymatic chemical process. In addition, environmental (the absence of large industrial factories) and seasonal climatic characteristics of our region (West Ukraine) that can affect the concentration of toxic glycation agents in the air should be taken into account.

In our previous work on Alzheimer's disease and vascular dementia, there were statistically significant diminished levels of AGE10 compared to those of the healthy group in the same age. In the same patients, enhanced levels of immune complexes AGE10-anti-AGE10 could be determined. We have speculated on the possible accumulated-AGE10 immune complexes in tissues as the most likely mechanism of these diseases [18]. It should be noted that synthetic MAGE compounds are obtained in anhydrous conditions and are different than conventional compounds formed in aqueous conditions; thus, AGE10 epitope does not cross-react with other known advanced glycation end products [14].

It has been shown in the present study that healthy individuals have significantly more fluorescent AGEs. The question why allergic rhinitis patients have lower levels of determined AGEs remains unanswered. Perhaps the cells of the immune system that are continuously activated in these patients effectively remove glycation products from the body. This is our hypothesis and it requires thorough research. The other explanation is the presence of immune complexes, as revealed in this study. In allergic patients with chronic EBV infection-elevated levels of immune complexes can be explained by the formation of immune complex type of allergic reaction. This type is characterized by tissue damage by immune complexes. The formed antibodies belong mainly to the IgG and IgM classes. It is understood that antibodies are directed against viral antigens [22, 44–46]. Here, we observe immune complexes containing AGE10 and anti-AGE10 constituents. In the development of inflammation, the deposition of immune complexes in the tissues is essential. Tissue destruction is associated with the action of platelets (blood clots formation) and neutrophils (release of enzymes and free oxygen radicals). After the activation, release proinflammatory cytokines IL1, IL-6, and IFN-*γ*, a platelet-derived growth factor (PDGF) engaging the formation of reactive oxygen intermediates (ROI). ROI oxidizes the lipids to generate peroxides and aldehydes. These lipid peroxidation products significantly oxidize lipid-derived aldehydes, are much more stable than the parent ROI and, therefore, can diffuse from their generation site and inflict damage at remote locations [47]. Excess cellular levels of ROI can lead to the activation of cell death processes such as apoptosis [48]. ROI promotes the formation of aldose reductase that catalyzes the reduction of aldehydes and carbonyls, including monosaccharides. These selected mediators further contribute to the deposition of immune complexes. Besides ROI, reactive nitrogen species (RNS) are involved in tissue destruction, forming nitrotyrosine, a product of tyrosine nitration mediated by reactive nitrogen species such as peroxynitrite anion and nitrogen dioxide. Nitrotyrosine is identified as an indicator or marker of cell damage, inflammation, and NO (nitric oxide) production [49].

On the other hand, macrophages excrete matrix metalloproteinases (MMPs). They are involved in the cleavage of cell surface receptors, the release of apoptotic ligands (such as the Fas ligand), and chemokine/cytokine inactivation [50]. Therefore, in places of deposition of immune complexes, the destruction of tissues is amplified.

Finally, it is observed that change in glycation levels occurs in patients with intermittent AR with chronic EBV infection at different stages of persistence during the exacerbation of AR. Our assumptions need to be validated on a larger patient population during exacerbation of intermittent AR.

## 5. Conclusions

No significant difference in the levels of AGEs determined by immunoenzymatic method in the patients' blood with intermittent AR and chronic EBV persistence in the active and latent phases was detected. However fluorescent components are in low amounts in the patient groups. Differentiation of patient groups is more clearly visible when specific immune complexes are determined, especially in active state where the amount of immune complexes is distinctly higher than in latent state patients and in healthy control. Therefore, measuring the amounts of specific immune complexes could be a good candidate for a marker of the active state of disease. The studies were performed during the period of AR remission; therefore, the question should be further investigated whether EBV in the active as well as in the latent phases of infection affect the glycation level in patients with intermittent AR. One can speculate that active state promotes immune complexes formation. Thus, determination of AGE10, the immune complexes, and the fluorescence may in a better way reflect the status of the disease. These parameters should be investigated in patients during also the other periods with intermittent AR to further observe their diagnostic value. However, we do not exclude the influence of allergic inflammation factors on the level of glycation in these patients during the exacerbation of intermittent AR, besides the presence of specific immune complexes.

## Figures and Tables

**Table 1:** Correlations between studied parameters.

		AGE10 (*μ*g/mL)	AGE10 in IC (*μ*g/mL)	Pentosidine (a.u.)	AGE total (a.u.)
AGE total in serum (a.u.)	*r*	**0.5019**	-0.2123	**0.6952**	
	*p*	**0.002**	0.228	**001**	
Pentosidine in serum (a.u.)	*r*	0.2276	-0.2659		**0.6952**
	*p*	0.195	0.129		**001**
AGE10 in IC (*μ*g/mL)	*r*	**-0.3901**		-0.2659	-0.2123
	*p*	**0.023**		*p* = 0.129	0.228
AGE10 in serum (*μ*g/mL)	*r*		**-0.3901**	0.2276	**0.5019**
	*p*		**0.023**	*p* = 0.195	**0.002**

Statistically significant correlations are marked in bold.

## Data Availability

The data that support the findings of this study are available from the first author [S.Z., svitlanazu@gmail.com] upon reasonable request.
